# Adenotonsillar Hypertrophy and Cardiopulmonary Status: A Correlative Study

**DOI:** 10.7759/cureus.31175

**Published:** 2022-11-06

**Authors:** Prasad Deshmukh, Puja Lakhotia, Sagar S Gaurkar, Aditya Ranjan, Manisha Dash

**Affiliations:** 1 Otolaryngology-Head and Neck Surgery, Jawaharlal Nehru Medical College, Datta Meghe Institute of Medical Sciences, Wardha, IND; 2 Otolaryngology-Head and Neck Surgery and Surgical Oncology, Jawaharlal Nehru Medical College, Datta Meghe Institute of Medical Sciences, Wardha, IND

**Keywords:** correlation pft adenotonsillar hypertrophy, correlation ecg adenotonsillar hypertrophy, endoscopic grading adenotonsillar hypertrophy, adenotonsillar hypertrophy, ecg, pft

## Abstract

Introduction

Adenotonsillar hyperplasia in childhood is a common phenomenon. It has been reported in the literature that increased upper-airway resistance resulting from hypertrophied tonsils and adenoids can cause intermittent airway obstruction, chronic alveolar hypoventilation, and even lead to severe cardiopulmonary complications such as right ventricular (RV) failure and cor-pulmonale, a near-lethal culmination of pulmonary artery hypertension (PAH). This study was undertaken to explore and examine the association of adenotonsillar hypertrophy and cardiopulmonary status in patients with complaints of upper airway obstruction below the age of 20 years and to analyze the effect of adenotonsillar enlargement on pulmonary function tests and cardiac aberration reflected in the electrocardiography (ECG) changes.

Methods

This study included patients visiting or admitted to the Otolaryngology/Ear, Nose, and Throat (ENT) and Paediatrics department of Acharya Vinoba Bhave Hospital, Sawangi (Meghe), Wardha, Maharashtra, India. It is an Observational Prospective Study conducted on 75 Patients (50 cases and 25 controls) below the age of 20 years. Inclusion criteria for cases included all patients of adenoid hypertrophy (AH) and adenotonsillar hypertrophy (ATH). Inclusion criteria for controls comprised all patients with a history and clinical examination not suggestive of any upper airway and pulmonary disease. All the patients were subjected to pulmonary function tests (PFT) and electrocardiography (ECG), and the values were compared.

Results

It was found that there was a decrease in the value of the parameters of the pulmonary function tests (PFT), which includes forced expiratory volume in the first second (FEV1), forced vital capacity (FVC), the ratio of the forced expiratory volume in the first one second to the forced vital capacity of the lungs (FEV1/FVC) and peak expiratory flow rate (PEFR) with increasing severity of the endoscopic grades of adenoid hypertrophy. This was found to be statistically significant. All the PFT parameters were significantly lower in the subset of patients with ATH compared to those with AH only, emphasizing the compounding effect of tonsillar volume. Between case and control subjects also, these differences were statistically significant. Seventeen (34%) out of the 50 patients studied in the present work were found to have abnormalities in their ECG, while no subject in the control group had any departure from normal. Nine of these 17 patients had AH, and eight had the adenotonsillar disease. In the 9 patients (18%) with AH, sinus arrhythmia was seen in 5 (10%), sinus tachycardia in 3 (6%), and Mobitz type 1 block in 1 (2%) patients. In 8 patients (16%) with AH, sinus arrhythmia was seen in 4 (8%), sinus tachycardia in 3 (6%), and Mobitz type 1 block in 1 (2%) patients. Overall, sinus arrhythmia was the commonest finding seen in 9 patients (18%).

Conclusion

Chronic obstructive adenotonsillar hypertrophy causes significant cardiovascular and pulmonary changes, which is often overlooked in the clinical setting. Symptoms of progressive pulmonary hypertension are minimal until the rapid onset of severe cardiac decompensation occurs. These entire cascades of events are reversible in the early stages and thus mandate early detection and treatment. Performing PFT and ECG in children with adenotonsillar disease is not mandatory but profitable even in the absence of obvious symptoms of upper airway obstruction.

## Introduction

Adenotonsillar hypertrophy in childhood is a common phenomenon. Nasopharyngeal tonsils and palatine tonsils, tubal tonsils, and lingual tonsils are members of the Waldeyer Ring, named after the 19^th^-century German anatomist, Sir Heinrich Wilhelm Gottfried Waldeyer, who first described it in 1884 [1,2]. Generally, these lymphoid tissues encircling the upper airway and food passage show a remarkable and progressive increase in size till the age of approximately 12 years and then involute in adolescence and adulthood. This can be ascribed to their immunological role in early life. Adenotonsillar hypertrophy (ATH) is the most common cause of upper airway obstruction (UAO) and obstructive sleep apnea syndrome in children [3]. A series of observations in the last decade has provided a possible example of the effects of nasopharyngeal obstruction on the heart and lungs [3,4]. It has been reported in the literature that increased upper airway resistance resulting from hypertrophied tonsils and adenoids can cause intermittent airway obstruction, chronic alveolar hypoventilation, and even lead to severe cardiopulmonary complications such as right ventricular (RV) failure and cor-pulmonale which is a near-lethal culmination of pulmonary artery hypertension (PAH) [4]. This study was undertaken to explore and examine the association of adenotonsillar hypertrophy and cardiopulmonary status in patients with complaints of upper airway obstruction below the age of 20 years. In all 50 patients, grading of adenoids and tonsils was done clinically, and endoscopically, pulmonary function was assessed by spirometry, and electrocardiography (ECG) recording was done. The principle aim of this study is to analyze the effect of adenotonsillar enlargement on pulmonary function tests and ECG reflecting cardiac aberrations.

## Materials and methods

This study was conducted on patients visiting the outpatient department or admitted in the wards of the department of Otolaryngology/Ear, Nose and Throat (ENT) and Paediatrics of Acharya Vinoba Bhave Hospital, Sawangi (Meghe), Wardha, Maharashtra State, India. It is an observational prospective study conducted on 75 patients (50 cases and 25 controls) below the age of 20 years. Inclusion criteria for cases included all patients of adenoid hypertrophy (AH) and adenotonsillar hypertrophy (ATH) up to 20 years of age. Inclusion criteria for controls comprised all patients with a history and clinical examination not suggestive of any upper airway and pulmonary disease. Exclusion criteria for cases comprised of all patients of AH and ATH above 20 years of age, adenotonsillar hypertrophy with acute exacerbation, isolated tonsillar hypertrophy, patients with cardiopulmonary diseases, patients with nasal polyps, deviated nasal septum or any structural abnormality of the thoracic cage affecting pulmonary function, primary pulmonary hypertension, systemic diseases, obesity, craniofacial anomaly, and genetic syndrome and patient not consenting. Exclusion criteria for controls included all patients with any cardiopulmonary diseases.

The study duration was from September 2015 to September 2017. Fifty patients less than or equal to 20 years of age with signs and symptoms suggestive of adenoid or adenotonsillar hypertrophy were selected for the study. After a thorough and careful ENT examination, patients were subjected to endoscopic assessment, and grading of adenoid and adenotonsillar hypertrophy was done. Photographic documentation of various modalities of assessment of adenoid size was also done. Once the diagnosis was established, patients were further subjected to two major investigations: Pulmonary function test/spirometry and electrocardiography (ECG). ECG was done per 25 patients less than or equal to 20 years of age fitting within the above-mentioned selection criteria and was accrued as controls for the study. All the patients were subjected to pulmonary function test/spirometry and ECG.

Ethical declaration

All procedures performed in this study involving human participants were in accordance with the ethical standards of the internal Institutional Ethics Committee, Jawaharlal Nehru Medical College and Acharya Vinoba Bhave Rural Hospital, Sawangi (Meghe), Wardha, Maharashtra, India (established under section three of the UGC Act vide Notification Number F-9-48/2004 - U3 Govt of India), with reference number DMIMS(DU)/IEC/2015-16/1636 approved on 26th of September, 2015. The approval has been granted on the assumption that the proposed work will be carried out in accordance with the ethical guidelines prescribed by Central Ethics Committee on Human Research (CECHR). 

Pulmonary function test/Spirometry

Spirometric measurements were carried out on an RMS Helios 401 (Recorders & Medicare Systems Pvt. Ltd, India), a spirometer, in a sitting position with the nose clipped. The patient was asked to make a series of forced expirations from the position of full inspiration into the mouthpiece of the spirometric system and a series of forced inspirations starting from the position of full expiration. These maneuvers were repeated until three closely matching forced expirations and inspirations were recorded.

Electrocardiogram

Twelve-lead ECG was performed on all the patients diagnosed as having adenoid/adenotonsillar hypertrophy using the BPL AR 2100 (Royal Health Care, Mumbai, India) machine. The parameters measured on the ECG included the heart rate, rhythm, characteristics of P, QRS, and T waves, and the presence of evidence of atrial hypertrophy, ventricular hypertrophy, and axis deviation. The ECG was read by a single experienced cardiologist. The findings were recorded under two headings - significant findings or normal ECG.

Observations

Data gathered was entered in the proforma meant for the study and was statistically analyzed.

Informed Consent

The procedure was explained in detail to the parents, and written informed consent was obtained before enrollment.

Clinical Grading of Tonsils

All 50 patients were clinically examined for the presence of significant tonsillar hypertrophy, i.e., hypertrophy >2+, as per the assessment scale described by Brodsky (Table 1) [5]. 

**Table 1:** Clinical grading of tonsils

Grade	Features
+0	Tonsils are situated in the tonsillar fossa
+1	Tonsils sit just outside of the tonsillar fossa with obstruction of less than 25 percent of the airway
+2	Tonsils are readily seen in the airway: 25 to 50 percent of the airway is obstructed
+3	Tonsils causing 50 to 75 percent obstruction of the airway
+4	Tonsils involve a greater than 75 percent obstruction of the airway

Endoscopic Grading of Adenoids

All the patients were subjected to post nasal space examination using nasopharyngeal examination by the rigid endoscope, Karl Storz zero-degree nasal endoscope (2.7 mm), made of stainless steel body was used. Endoscopy was done after treating the nose with 4% lidocaine mixed with adrenaline in both nostrils. The posterior end of the middle turbinate was considered a fixed anatomical landmark for viewing posterior choanae and adenoid hypertrophy. Adenoids were graded based on the classification given by Cassano as follows (Table 2) [6].

**Table 2:** Endoscopic grading of adenoids

Grade	Features
1	Choanal adenoid, occupying upper segment of nasopharynx (<25% of the choana)
2	Adenoid tissue occupying upper half of nasopharynx (25% to < 50%)
3	Adenoids extending over the nasopharynx obstructing the choana and partially the tube (50% to <75%)
4	Total choanal obstruction (≥75%)

Statistical analysis

Statistical analysis was done by using descriptive and inferential statistics using the chi-square test, student's unpaired t-test, regression analysis, one-way analysis of variance (ANOVA), and Kappa Statistics and software used in the analysis were IBM Corp. Released 2011. IBM SPSS Statistics for Windows, Version 20.0. Armonk, NY: IBM Corp. and GraphPad Prism 7.0 version and probability (p-value) were calculated.

## Results

A total of 75 patients were enrolled in the study comprising 50 cases and 25 controls. Maximum patients with only adenoid hypertrophy (AH) as well as with adenotonsillar hypertrophy (ATH) were between the age group of 6 and 10 years (18, 36% (AH) and 13, 26% (ATH), respectively). The mean age of the patients was 10±3.09 years, while in the control group, it was 9.88±2.20 years. The mean age of patients with AH was 10.33±3.63 years, and with ATH was 9.50±1.98 years (Table 3). 

**Table 3:** Distribution of patients according to age AH: Adenoid hypertrophy, ATH: Adenotonsillar hypertrophy

Age Group(years)	Case Group		Control Group
	AH	ATH	
0-5 years	2(4%)	0(0%)	0(0%)
6-10 years	18(36%)	13(26%)	15(60%)
11-15 years	8(16%)	7(14%)	10(40%)
16-20 years	2(4%)	0(0%)	0(0%)
Total	30(60%)	20(40%)	25(100%)
Mean ±SD	10.33±3.63	9.50±1.98	9.88±2.20
Range	5-19 years	6-13 years	6-14 years
	10±3.09 (5-19 years)		

Males (28, 56%) marginally outnumbered females (22, 44%) with a male-to-female ratio (M:F ratio) of 1.27:1. In AH only patients, males and females were equal in number (15 each, 30%) with an M:F ratio of 1:1 while in patients of ATH males (13, 26%) were almost twice as females (7, 14%), the M:F ratio being 1.85:1. Table 4 shows the gender-wise distribution of patients.

**Table 4:** Distribution of patients according to gender AH: Adenoid hypertrophy, ATH: Adenotonsillar hypertrophy

Gender	Case Group		Control Group
	AH	ATH	
Male	15(30%)	13(26%)	18(72%)
Female	15(30%)	7(14%)	7(28%)
Total	30(60%)	20(40%)	25(100%)

The patients were also analyzed based on their geographical background and observed that the maximum number of patients (31, 62%) belonged to the urban area while 19 (38%) hailed from the rural area (Table 5) and out of a total of 50 patients studied, 30 (60%) had AH, and 20 (40%) had ATH (Table 6).

**Table 5:** Geographic distribution of patients

	Area		Total (n=50)
	Rural	Urban	
Adenoid Hypertrophy (AH)	13 (26%)	17 (34%)	30 (60%)
Adenotonsillar Hypertrophy (ATH)	6 (12%)	14 (28%)	20 (40%)
Total (n=50)	19 (38%)	31 (62%)	50 (100%)

**Table 6:** Distribution of patients according to site of obstruction

Site of Obstruction	No of patients	Percentage
Adenoid Hypertrophy (AH)	30	60
Adenotonsillar Hypertrophy (ATH-Patients with Grade III and IV tonsils as per the Brodsky classification)	20	40
Total	50	100

The presenting symptoms of the patients with their frequency are displayed in Table 7. In patients with adenoid hypertrophy, the commonest presenting symptom was mouth breathing (23, 46%) followed by snoring (14, 28%) and earache (10, 20%). In patients with adenotonsillar hypertrophy, the commonest presenting symptoms were mouth breathing and snoring (17, 34% each), followed by obstructive symptoms causing disturbed sleep at night (6, 12%). Overall, mouth breathing (40, 80%), snoring (31, 62%), and earache (11, 22%) were the common manifestations.

**Table 7:** Symptoms of patients with adenoid and adenotonsillar hypertrophy AH: Adenoid hypertrophy, ATH: Adenotonsillar hypertrophy

Symptoms	No. of Patients		Total
	AH	ATH	
1. Mouth Breathing	23 (46%)	17 (34%)	40 (80%)
2. Snoring	14 (28%)	17 (34%)	31 (62%)
3. Disturbed sleep	2 (4%)	6 (12%)	8 (16%)
4. Ear discharge	7 (14%)	1 (2%)	8 (16%)
5. Ear ache	10 (20%)	1 (2%)	11 (22%)
6. Daytime Hypersomnolence	1 (2%)	0 (0%)	1 (2%)
7. Nasal discharge	8 (16%)	2 (4%)	10 (20%)

Nasal endoscopy to grade adenoids was carried out, as per the guidelines given by Cassano [6]. Adenoid enlargement was graded depending on the percentage of choanal obstruction with the nasal endoscope (0 degrees, 2.7mm) placed at the posterior end of the middle turbinate. Overall, most patients had grade 3 AH (26, 52%). In patients with only AH, a maximum number of patients were observed in grade 4 (13, 26%), followed by grade 3 (11, 22%). While in patients with ATH, grade 3 enlargement (15, 30%) was most commonly observed. This is shown in Table 8.

**Table 8:** Endoscopic grading of patients with adenoid and adenotonsillar hypertrophy AH: Adenoid hypertrophy, ATH: Adenotonsillar hypertrophy

Endoscopic grade	AH	ATH	Total
Grade 1	1 (2%)	--	1 (2%)
Grade 2	5 (10%)	1 (2%)	6 (12%)
Grade 3	11 (22%)	15 (30%)	26 (52%)
Grade 4	13 (26%)	4 (8%)	17 (34%)
Total	30	20	50 (100%)

The mean of the parameters of the pulmonary function test (PFT), which includes forced expiratory volume in the first second (FEV1), forced vital capacity (FVC), the ratio of the forced expiratory volume in the first one second to the forced vital capacity of the lungs (FEV1/FVC) and peak expiratory flow rate (PEFR) was studied with respect to the endoscopic grade of disease. Patients with AH: The values of FVC, FEV1 and PEFR were noted in grades 1, 2, and 4 AH, and the mean value of the aforementioned parameters was noted is given in Table 9. Patients with ATH: All four PFT parameters viz. FVC, FEV1, FEV1/FVC, and PEFR were noted along with the endoscopic severity of the disease, and the p-value was calculated as shown in Table 9. AH vs. ATH: The PFT values of the two subsets of patients were compared (Table 9).

**Table 9:** Comparison between endoscopic grade and PFT values AH: Adenoid hypertrophy, ATH: Adenotonsillar hypertrophy, FVC(L): Forced vital capacity in liters, FEV1(L): Forced expiratory volume in the first second in liters, FEV1/FVC: Ratio of forced expiratory volume in first second to forced vital capacity, PEFR(L/s): Peak expiratory flow in liters per second, f- value: Ratio of two variances, or technically, two mean squares, p-value: Probability value, S: Significant, NS: Not significant

Table 9: PFT vs. ENDOSCOPIC GRADING								
Endoscopic Grade	AH				ATH			
	FVC (L)	FEV_1_ (L)	FEV_1_/FVC	PEFR (L/s)	FVC (L)	FEV_1_ (L)	FEV_1_/FVC	PEFR (L/s)
Grade 1	1.94±0	1.78±0	91±0	2.76±0	-	-	-	-
Grade 2	1.23±0.58	1.08±0.56	86.68±4.92	2.15±0.55	0.93±0	0.77±0	82.80±0	1.55±0
Grade 3	1.34±0.81	1.12±0.70	81.31±4.97	2.22±0.49	0.88±0.20	0.67±0.20	75.17±6.09	1.75±0.57
Grade 4	0.92±0.29	0.71±0.25	75.88±7.73	1.73±0.57	0.78±0.19	0.58±0.10	73.11±1.85	1.56±0.31
F-value	1.64	2.24	4.75	2.40	19.15	27.36	1.20	4.929
p-value	0.20,NS	0.10,NS	0.009,S	0.090,NS	<0.0001,S	<0.0001,S	0.32,NS	0.0017,S

Seventeen (34%) out of the 50 patients studied in the present work were found to have abnormalities in their ECG, while no subject in the control group had any departure from normal (Table 10). Table 10 displays the frequency and type of abnormality found in the ECG of patients with the adenoid and adenotonsillar disease. Nine of the 17 patients had AH, and eight had the adenotonsillar disease. In the nine patients (18%) with AH, sinus arrhythmia was seen in five (10%), sinus tachycardia in three (6%), and Mobitz type 1 block in one (2%) patient. In eight patients (16%) with AH, sinus arrhythmia was seen in four (8%), sinus tachycardia in three (6%), and Mobitz type 1 block in one (2%) patient. Overall, sinus arrhythmia was the commonest finding seen in nine patients (18%).

**Table 10:** Abnormal ECG findings in patients with adenoid and adenotonsillar hypertrophy AH: Adenoid hypertrophy, ATH: Adenotonsillar hypertrophy

ECG Findings	AH	ATH	Total (n=50)
Sinus Tachycardia	3 (6%)	3 (6%)	6 (12%)
Sinus Arrhythmia	5 (10%)	4 (8%)	9 (18%)
Mobitz Type 1 Block	1 (2%)	1 (2%)	2 (4%)
Total	9 (18%)	8 (16%)	17 (34%)

## Discussion

Sleep-disordered breathing (SDB) has an estimated prevalence of 11% in children [7]. Obstructive sleep apnea affects 2% of the pediatric population and is the most severe form of SDB [3]. Increased upper airway resistance during sleep is due to a combination of soft tissue hypertrophy, craniofacial dysmorphology, neuromuscular weakness, or obesity. Adenotonsillar hypertrophy is the most common cause of pediatric upper airway obstruction and can cause a multitude of cardiovascular and pulmonary complications like sleep apnea, sleeping disorders, pulmonary hypertension, cor-pulmonale, and even heart failure [8]. These complications are silent in their progression, and timely intervention results in complete reversal [8].

Detailed history and symptoms were elicited, followed by clinical examination and clinical grading and endoscopic grading of adenoids, ECG, and spirometry carried out, and observations emanating from the study were recorded and statistically analyzed. The mean age of adenoid hypertrophy (AH) and adenotonsillar hypertrophy (ATH) in the present study was 10±3.09 years. The mean age reported by most of the investigators agrees well with the present study [9-14]. In a study by Tatlipinar, the mean age for AH was 6.96±2.11 years, while for ATH was 6.96±1.68 years [15]. In the present study, the mean age of patients with AH was 10.33±3.63 years, while that with ATH was 9.50±1.98years. Exposure to the environment, to the pollutants, and most importantly, immunological stimulation coupled with allergic and inflammatory episodes makes this age group susceptible to AH and ATH.

Marginal male predominance was found in the present study. Variable preponderance has been reported by various investigators. Male predominance has been reported in some studies [10,12,13]. Female preponderance has been noted in another study [16]. Maximum patients (31, 62%) belonged to the urban area, while 19 (38%) hailed from the rural area. Rout et al. found an overwhelmingly high urban population (26/30, 87%) in their study, which aligns with the present study [17]. Niedzielska et al. observed that inhabitants of the towns reached poorer FVC results in comparison with those of rural areas [13]. It may result from air pollution or other factors and requires further research.

Out of a total of 50 patients with upper airway obstruction, 30 (60%) had AH, and 20 (40%) had ATH. Though not much literature is available on this dimension of the study, Tatlipinar et al. found that AH was seen in 40 patients (42%), and ATH was present in 41 patients (43%) [15]. The present study differs from this observation, but this being the only study available even after extensive search prevents us from drawing any firm inference regarding the predilection for the site of obstruction.

The range of symptoms in the present study was similar to that reported by other authors. Mouth breathing (80%) and snoring (62%) in general and disturbed sleep (16%) and nasal discharge (20%) in particular were observed less frequently in the present study. Racial factors, nasopharyngeal size, use of nasal decongestants, and variable significance attached to these otherwise important symptoms like disturbed sleep and nasal discharge may explain the less frequent reporting of these symptoms by the parents [18]. Nasal endoscopy to grade adenoids was carried out as per the guidelines given by Cassano [5]. In patients with only AH, a maximum number of patients observed had grade 4 (13, 26%) adenoid hypertrophy, followed by grade 3 (11, 22%). While in patients with ATH, grade 3 enlargement (15, 30%) of adenoids was most commonly observed. However, this difference was not found to be statistically significant (p<0.05). Patients with AH are mostly presenting with grade 4, while those with ATH are with grade 3. Seeking early medical help by patients with ATH could be due to the additive and compounding effect of enlarged tonsils.

Overall, in the present study, a maximum number of patients with upper airway obstruction had grade 3 enlargement (26, 52%), while the least number of patients (1, 2%) had grade I enlargement. The study conducted by Yaseen echoes similar findings as in their series of patients they observed maximum patients with grade 3 enlargement of 33.33% and least with grade 1 13.3% enlargement [19]. Nasal endoscopy has been considered the standard method for the assessment of adenoid size in several studies [20-23]. It provides a direct anatomical view of the nasopharynx for determining the size of the adenoid and the degree of obstruction of the choanal opening. Nasal endoscopy gives objective and highly accurate results that correlate more closely with the severity of the AH than the lateral neck X-ray [20,21,24,25]. In the present study, the mean of several PFT parameters was studied with respect to the endoscopic grade of the disease.

In patients with AH a successive decrease in FVC, FEV1 and PEFR was noted over endoscopic grades 1, 2, and 4, but a higher mean value of the aforementioned parameters was seen in grade 3, as compared to grade 2 AH. However, a consistent and statistically significant decreasing trend with increasing endoscopic severity of the disease was noted in FEV1/FVC with a p-value of 0.009. In patients with ATH, a progressive decrease in all four PFT parameters viz. FVC, FEV1, FEV1/FVC, and PEFR were noted with an increase in the endoscopic severity of the disease. This decrease was found to be statistically significant (p<0.05) for all parameters except FEV1/FVC. AH vs. ATH-On comparing the PFT values of patients AH and ATH-The corresponding parameters were found to be lower in patients of ATH than those with only AH.

The paucity of studies studying this facet constrains us from a comparison. All 50 patients and 25 controls were subjected to 12-lead ECG. Seventeen (34%) out of the 50 patients studied in the present work were found to have abnormalities in their ECG, while no subject in the control group had any departure from normal. Overall, sinus arrhythmia was the commonest finding seen in nine patients (18%). Norte noted that the use of ECG to diagnose pulmonary hypertension in infants with ATH associated with sleep apnea revealed low sensitivity [26]. Yılmaz evaluated the prevalence of arrhythmias, heart rate variability (HRV), and heart rate turbulence (HRT) using 24-hour Holter ECG monitoring pre and postoperatively in children with ATH [27]. They found that although some ECG and Holter findings, such as sinus tachycardia and Mobitz type 1 second-degree atrioventricular block, improved after the operation, the prevalence of arrhythmias and HRV and HRT values did not change significantly in the postoperative period. In a study by Fasunla, seven (9.46%) patients had abnormal ECG findings, of which three patients had isolated right ventricular hypertrophy, one patient had right atria hypertrophy, and another had right axis deviation, and the remaining two patients had bilateral ventricular hypertrophy [28]. Rosenzweig states that the ECG commonly shows right atrial enlargement, right-axis deviation, and right ventricular hypertrophy with secondary T-wave changes; however, these findings do not necessarily parallel the severity of the underlying pulmonary hypertension [29]. This area needs further research.

Chronic obstructive adenotonsillar hypertrophy causes significant cardiovascular and pulmonary changes. Recent studies have suggested that chronic pulmonary disorders in adolescents and adults have their origin in the first few years of life and may be related to chronic upper airway obstruction [30]. Unfortunately, this is often overlooked in the clinical setting. The symptoms of progressive pulmonary hypertension are minimal until the rapid onset of severe cardiac decompensation occurs. These entire cascades of events are reversible in the early stages and thus mandate early detection and treatment. Performing PFT, ECG, and Doppler echocardiographic examination in children with adenotonsillar disease is not mandatory but profitable even in the absence of obvious symptoms of upper airway obstruction [30]. Current literature supports performing PFT in children as young as 5-6 years as their development helps them to respond to given commands. As every child with ATH carries the risk of obstructive lung disease and/or pulmonary hypertension, the complementary, comprehensive, and correlative role of clinical, endoscopic, and radiological assessment cannot be over-emphasized.

The changes seen in PFT can be based on the fact that the respiratory system shares a unified airway with the same mucosal carpet hence showing common diseases. ECG was done in the patients purely out of the examiner's interest to see incidental subclinical and subtle findings as respiratory comprise is directly linked with cardiac activity alterations and any changes in the early decade which is considered physiological can lead to pathological conditions if it persists for a long term. 

The inadequate sample size is the limitation of this study. The period for which the patient had adenoid or adenotonsillar hypertrophy was not taken into account. Cardiopulmonary parameters other than pulmonary function tests and electrocardiograms like echocardiograms could be taken in further studies. Similar studies are needed to be conducted on larger groups with more parameters to establish the impact of adenoid hypertrophy and adenotonsillar hypertrophy on cardiopulmonary status.

## Conclusions

Chronic obstructive adenotonsillar hypertrophy causes significant cardiovascular and pulmonary changes. Unfortunately, this is often overlooked in the clinical setting. The symptoms of progressive pulmonary hypertension are minimal until the rapid onset of severe cardiac decompensation occurs. These entire cascades of events are reversible in the early stages and thus mandate early detection and treatment. Performing PFT and ECG in children with adenotonsillar disease is not mandatory but profitable even in the absence of obvious symptoms of upper airway obstruction. Through this study, we attempt to throw light on the often-missed consequence of AH and ATH, which might help in counseling regarding surgical interventions required.
